# Old and outdated radiology equipment in Croatia—radiation safety and economic consequences

**DOI:** 10.1007/s13244-016-0464-y

**Published:** 2016-02-16

**Authors:** Iva Busic Pavlek, Zoran Brnic, Sasa Schmidt, Tomislav Krpan, Ivana Kralik

**Affiliations:** Department of Diagnostic and Interventional Radiology, University Hospital Centre Sestre Milosrdnice, Vinogradska cesta 29, Zagreb, 10000 Croatia; State Institute for Radiological and Nuclear Safety, Zagreb, Croatia

Dear Editor,

Inspired by the ESR position statement on the renewal of radiological equipment and the worsening conditions of the equipment we use daily, we decided to send you this letter to raise expert public attention concerning this burning issue. With regards to the ESR position statement [1], which adopted general rules endorsed by The Canadian Association of Radiologists [2] regarding the life cycle of various types of equipment, we analysed the current state of Croatian radiological equipment, which makes the largest contribution to the public’s radiation exposure.

*Main Messages*

• *Ionising radiation from medical imaging contributes significantly to the population’s radiation exposure*

• *Technological innovations enable dose reduction, thus lowering the chance of adverse effects*

• *None of the analysed equipment modalities in Croatia fulfills the requirements for reasonable renewal*

• *Using up-to-date equipment can ensure that the benefits of radiological procedures outweigh the risks*

It is recommended that at least 60 % of the installed equipment in radiology departments be up to 5 years old. Up to 30 % should be 6–10 years old, whereas no more than 10 % of the equipment should be older than 10 years.

Technological innovations are helping us reduce the ionising radiation dose delivered to patients and also provide better image quality, thus improving diagnoses and treatments.

We statistically analysed data on the number and age of the devices installed in Croatia for radiological diagnostic and therapeutic procedures (CT, angiography, mammography) obtained from Registry of Radiological Equipment in the State Institute for Radiological and Nuclear Safety and compared them with those from other European countries [3].

The age structure of angiography and CT equipment is given in Tables 1 and 2.

**Table 1:** Age structure of angiography equipment in the surveyed countries compared to the European average

Country	Croatia	Serbia	Romania	Germany	UK	Europe
Age (years)						
0–5	46.6 %	40 %	33 %	47 %	40 %	42 %
5–10	10 %	55 %	62 %	30 %	42 %	37 %
>10	43.4 %	15 %	5 %	23 %	18 %	21 %

**Table 2:** Age structure of CT equipment in the surveyed countries compared to the European average

Country	Croatia	Serbia	Romania	Germany	UK	Europe
Age (years)						
0–5	22 %	35 %	66 %	50 %	45 %	50 %
5–10	32.5 %	45 %	29 %	39 %	45 %	38 %
>10	45.5 %	20 %	5 %	11 %	10 %	12 %

Mammography units in Croatia can be classified according to age as follows: 17 % 0–5 years old, 21 % 5–10 years old, and 62 % more than 10 years old.

Croatia has 43.4, 45.5, and 62 % outdated angiography, CT, and mammography equipment, respectively. Among the surveyed countries, this is the highest percentage of outdated equipment in all three modalities.

Devices for radiation exposure measurement and display are commonly lacking in the older equipment; hence, the radiation exposure level is frequently unknown. This might lead to delays in the diagnosis and treatment of patients or radiation overexposure of both the patients and medical staff. Unreasonably high patient doses pose a particular problem in screening mammography, especially if adequate image quality is not reached. The problem is not simply the age of the equipment used. New technological breakthroughs render some equipment obsolete. If taking into account that for each year of service the estimated cost of maintenance is 5–6 % of the price of a new device [4], it is easy to calculate that outdated equipment is actually quite expensive. Pricy servicing of old equipment severely affects both public and private clinics, increasing the price per procedure on the market.

We as experts should develop control over the quality of the equipment used. We need to put pressure on decision makers to develop a comprehensive plan for renewal of radiological equipment. Only in this manner can we guarantee our users that the benefits of radiological procedures outweigh the risks.
